# Historical Plant Embryos as Alternative Sources of Ancient DNA for Whole Genome Sequencing

**DOI:** 10.1111/1755-0998.70210

**Published:** 2026-10-07

**Authors:** Hong Phuong Le, Stefano Porrelli, Yoon Kyung Lee, Simon Juraver, Flora Pennec, Mark Nesbitt, Koji Numaguchi, Rafal Marek Gutaker

**Affiliations:** ^1^ Royal Botanic Gardens Kew Richmond, Surrey UK; ^2^ Department of Agriculture, Forestry and Bioresources, Plant Genomics and Breeding Institute, Research Institute for Agriculture and Life Sciences Seoul National University Seoul South Korea; ^3^ Direction Générale Déléguée Aux Collections Muséum National D'histoire Naturelle Paris France; ^4^ UMR 7206 Eco‐Anthropologie (CNRS‐MNHN‐Université Paris Cité) Paris France; ^5^ Graduate School of Agricultural Science Kobe University Kobe Japan

**Keywords:** ancient DNA, genomic resources, herbarium, plant microevolution

## Abstract

Natural history and agricultural collections contain hundreds of millions of specimens classified in terms of time, space and taxonomy. Since the first success of ancient DNA (aDNA) isolation in the 1980s, these repositories, including herbaria for plants, have been intensively used to support studies in taxonomy, macroevolution and genetic responses to anthropogenic activities. For herbarium specimens, aDNA is usually extracted from leaf samples and is highly fragmented (typically length of 50–100 bp). To optimise the amount of data retrieved and minimise destructive sampling, we isolated DNA from an unconventional plant tissue type—seed embryos. We carried out whole‐genome sequencing and compared sequenced DNA quality between embryo and leaf tissue from the same specimen. We evaluated endogenous DNA proportion, median fragment length, damage fraction per site (*λ*), decay rates (*k*), nucleotide misincorporations and library complexity for three species: cultivated rice *Oryza sativa* , wild rice *O. rufipogon* and wild barley *Hordeum spontaneum* . In *O. sativa* , embryos exhibited significantly higher endogenous content and median fragment length than leaves. The superior DNA preservation was likely due to the protective role of the seed husk, which might play an important role in DNA preservation in plants collected in the tropics. By contrast, in temperate *H. spontaneum* , tissue type had minimal impact on DNA quality. Despite the minuscule size of the embryos, all derived genomic libraries were highly complex, sufficient for deep whole genome sequencing. These results highlight seed embryos as a promising alternative aDNA source for herbarium specimens, economic botany and anthropological museum collections.

## 1 Introduction

Herbarium specimens—pressed, dried plants mounted on paper—represent one of the largest and most comprehensive biological archives available for research. Globally, nearly 4800 herbaria house over 400 million specimens, encompassing all known plant taxa from all continents (*Index Herbariorum—The William & Lynda Steere Herbarium*, n.d.). These collections are invaluable for a wide range of research areas, including taxonomy, biodiversity and morphology (Heberling et al. 2019). With advances in molecular biology and next‐generation sequencing (NGS), herbaria, along with other historical plant collections, have become increasingly important as genetic resource repositories, supporting studies of plant evolution, phylogeny and ecological interaction (Burbano and Gutaker 2023; Kistler et al. 2020).

However, DNA isolated from herbarium specimens is reported to be highly fragmented and chemically damaged (Gutaker and Burbano 2017). Following plant collection and cell death, DNA is initially degraded by intracellular nucleases and microbial activities. Over time, hydrolytic and oxidative reactions further reduce DNA integrity at a slower pace (Lindahl 1993). DNA degradation and microbe colonisation occur during both the preparation process of herbarium specimens and storage. Post‐collection treatment of specimens sometimes involves heat for desiccation, freezing temperature for decontamination and in the past application of chemicals to prevent pest damage, which could exacerbate DNA degradation and damage (Brewer et al. 2019; Staats et al. 2011). The most common type of chemical damage in herbarium aDNA is cytosine deamination to uracil. While DNA in herbarium samples degrades 6–8 times faster than in archaeological bones of Moa birds, levels of cytosine deamination remain relatively low, reflecting its gradual, time‐dependent nature (Allentoft et al. 2012; Kistler et al. 2017, 2020; Porrelli et al. 2026; Weiß et al. 2016). Additionally, DNA from herbarium specimens is typically a complex mixture from the targeted plants and metagenomic DNA of microorganisms present before death or introduced post‐mortem, with the relative proportions of endogenous and exogenous DNA varying widely among samples (Gutaker and Burbano 2017; Ng and Gutaker 2025; Porrelli et al. 2026; Weiß et al. 2016).

Leaves have been the most used tissue for DNA isolation from herbarium specimens, due to their ready availability and potentially high DNA content (Drábková 2013; Särkinen et al. 2012). However, the quality and quantity of DNA isolated from herbarium leaves can vary widely among taxonomic groups and individual specimens, affected by factors including sample age, taxon and the source herbarium (Brewer et al. 2019; Kates et al. 2021; Porrelli et al. 2026; Quatela et al. 2023). Additionally, leaf tissue is not always present in sufficient quantity, particularly for small plants or specimens with few leaves. These limitations have prompted the development of non‐destructive sampling methods that minimise damage to irreplaceable material while still yielding enough endogenous DNA for downstream genomic analyses (Sugita et al. 2020).

Aside from leaves, many studies have investigated DNA isolation from non‐viable archaeological and historical seeds, including rice and barley seeds or seed remains (Hagenblad et al. 2024; Hiraoka et al. 2009; Kobayashi et al. 2006; Leino et al. 2009; Lister et al. 2009; Mascher et al. 2016; Muto et al. 2020; Palmer et al. 2009). Additionally, a few investigations have attempted DNA isolation from aged or mummified seed embryos, using either single embryos or pools of up to 16 embryos from certain eudicot and gymnosperm species (Rogers and Bendich 1985; Walters et al. 2006). The extent of DNA degradation and contamination is influenced by both sample age and species, while the seed coat may provide additional protection against DNA damage (Gugerli et al. 2005; Leino et al. 2009; Walters et al. 2006). In the singular case of large seeds from date palm, DNA was intact sufficiently to reportedly allow germination from a 2000‐year‐old archaeological sample (Gros‐Balthazard et al. 2021).

However, surrounding storage compounds such as carbohydrates, lipids and polyphenols can interfere with DNA isolation from whole seeds and reduce yield and purity (Liang et al. 2016). An additional complicating factor is that most seeds contain tissues of different individuals and of various ploidy levels, with maternal seed coat (2*n*), mixed endosperm (3*n*) and embryo (2*n*). Only the embryo represents the true diploid genotype of the progeny and consists of densely packed cells with relatively lower storage and secondary metabolite compounds, making them a potentially better target for DNA isolation from historical plant materials (Bennett et al. 1975; Itoh et al. 2005).

This study assessed the feasibility of isolating and sequencing DNA from single seed embryos from historical plant samples for whole genome sequencing. To that end, we compare DNA quality between embryo and leaf samples obtained from the same herbarium specimen across 26 specimens representing two species of rice ( *Oryza sativa* , *Oryza rufipogon* ) and one of barley ( *Hordeum spontaneum* ). We evaluated DNA preservation and sequencing performance using metrics such as endogenous DNA content, median fragment length, damage fraction per site, decay rates, nucleotide misincorporations and library complexity.

## 2 Materials and Methods

### 2.1 Herbarium Specimen Sampling

Plant materials (leaves and seeds) were obtained from herbarium specimens stored in the Ethnobotanical collection of National Museum of Natural History, France (NMNH) and Royal Botanic Gardens, Kew, United Kingdom (RBGK). *O. sativa* (cultivated rice) and *O. rufipogon* (wild rice) samples had been collected in Vietnam and Thailand, respectively, while *H. spontaneum* (wild barley) specimens had been collected in West Asia (Iraq, Jordan, Syria and Turkey) and Afghanistan. Ages of all samples ranged approximately from 30 to 200 years old (Supplementary Table 1).

### 2.2 DNA Isolation, Library Preparation and Sequencing

Rice and barley seeds obtained from herbarium specimens were selected based on maturity (plump, solid when pressed, no husk deformities) and then de‐husked, followed by embryo separation using Swann‐Morton sterile, single‐use stainless steel surgical scalpels (Fisher Scientific, 12,397,999). The separation took approximately 5 min per sample. Rice embryos were smaller and lighter than wild barley embryos (Figure 1).

**FIGURE 1:**
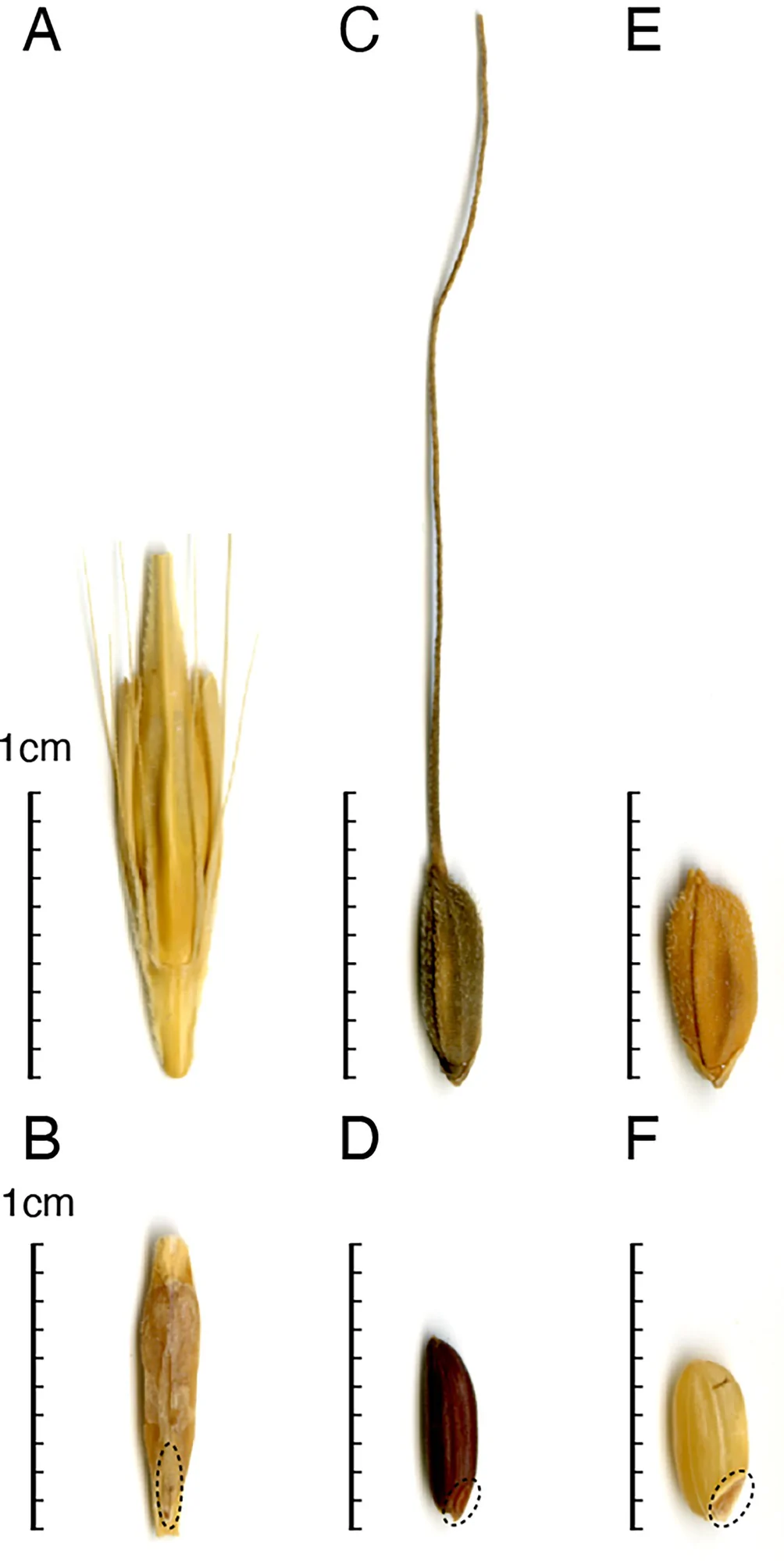
Representative husked and de‐husked seeds of *H. spontaneum* (A, B), *O. rufipogon* (C, D) and *O. sativa* (E, F) sampled from herbarium specimens, dated to 1885, 1929 and 1914, respectively. Dashed lines encircle embryos in the de‐husked seeds.

Dried tissues were subsampled into 2 mL PowerBead tubes (QIAGEN, 13117–50, metal 2.38 mm) and disrupted by using a Precellys Evolution homogeniser (2–4 cycles, soft program). Genomic DNA (gDNA) was isolated from dried leaf tissue (~4–17 mg) and a single seed embryo using the previously published *N*‐phenacylthiazolium bromide (PTB)—dithiothreitol (DTT) protocol (Gutaker et al. 2017; Latorre et al. 2020) in the clean room facilities at RBGK. In short, ground samples were lysed by resuspending in 1 mL of PTB lysis buffer (1% sodium lauryl sulphate (SDS), 10 mM Tris–HCl, 10 mM ethylenediaminetetraacetic acid (EDTA), 5 mM sodium chloride (NaCl), 2.5 mM PTB, 50 mM DTT and 0.4 mg/mL Proteinase K) and incubating at 37°C for 16–24 h in an incubated rotator. Lysate filtration and DNA purification were then carried out using a QIAGEN DNeasy Mini Plant kit (69106), following the manufacturer's manual. gDNA was eluted from the DNeasy spin column in 100 μL buffer AE and then quantified using a Quantus Fluorometer system (Promega). A negative control was included in each batch of DNA isolation.

Sequencing libraries were prepared from 20 or 26 μL of purified gDNA (depending on gDNA concentrations, Table 1) using previously published protocols (Kircher et al. 2012; Latorre et al. 2020; Meyer and Kircher 2010). Each batch of library preparation had a negative control (distilled water instead of DNA). The process consisted of four main steps (blunt‐end repair, adapter ligation, adapter fill‐in and indexing PCR). Uracil‐DNA glycosylase (UDG) treatment was not carried out during the blunt‐end repair process to preserve native levels of DNA damage patterns. Indexing oligo sequences were adapted from Meyer and Kircher (2010) and indexing PCR was run for 10–12 cycles. QIAGEN MinElute PCR Purification kit was employed to purify DNA after every step except for adapter fill‐in, where enzyme heat inactivation was performed. Indexed libraries were then eluted in 40 μL of Buffer EB, quantified and visualised by using Quantus Fluorometer and 4200 TapeStation (D1000, Agilent) systems.

**TABLE 1:** Yields of gDNA and sequencing libraries isolated from leaf or a single seed embryo sampled from *O. sativa* , *O. rufipogon* and *H. spontaneum* herbarium specimens.

Species	Sample ID	Collection year	Leaf			Seed embryo	
			Weight (mg)	gDNA yield (ng)	Library yield (ng)	gDNA yield (ng)	Library yield (ng)
*O. rufipogon*	HRG0844	1929	9.1	510	1600	3.49	28.08
	HRG0845	1929	9.7	275	640	5.04	86.4
	HRG0846	1929	9.5	461	440	BD	15.6
	HRG0847	1927	14.5	1100	2160	5.42	78.4
	HRG0848	1927	14.1	2100	1560	1.89	142.4
	HRG0855	1961	6.4	620	560	9.38	75.6
	HRG0858	1984	9.9	120	520	0.79	79.2
*O. sativa*	HRG0735	1914	13	78.4	6.52	6.79	12.2
	HRG0736	1914	15.2	19.6	8.64	8.55	13.76
	HRG0754	1914	15.1	104	9.4	3.37	10.92
	HRG0766	1914	15.5	9.06	8.52	5.41	82.8
	HRG0767	1914	15.5	44.4	9	47.4	170
	HRG0770	1914	16.2	19.4	12	6.96	75.2
	HRG0827	1869	16.8	119	680	0.15	51.6
	HRG0828	1869	12.8	6.55	119.6	12.6	145.2
	HRG0829	1869	13.9	19.9	19.36	3.49	68.4
*H. spontaneum*	HS0006	1966	6.2	680	1080	231	920
	HS0036	1994	7.1	1300	1400	790	840
	HS0046	1933	3.7	36.6	36.92	37.6	520
	HS0079	1943	3.6	178	199.2	2.31	37.68
	HS0084	1945	4.7	371	276	1000	720
	HS0093	1974	12	1300	840	760	720
	HS0103	1932	4.9	499	440	1300	760
	HS0138	1964	9	2200	440	700	1000
	HS0140	1885	10.4	115	600	344	1080
	HS0141	1822	6.1	346	520	97.6	920

Abbreviation: BD, below detection threshold.

Generated libraries were then pooled and sequenced on an Illumina NovaSeq X+ system at Macrogen Europe B.V using configuration for 2 × 150‐bp paired‐end sequencing.

### 2.3 Bioinformatics Analyses

Bioinformatic protocols for screening and determining properties of sequenced historical DNA libraries were adapted from Latorre et al. (2020) with some modifications, listed below.

#### 2.3.1 Read Processing and Alignment

Raw demultiplexed paired reads were merged (minimum overlap of 11 base pairs) and adapter sequence was removed using AdapterRemoval v2.3.4 (Schubert et al. 2016). Quality check of trimmed and merged reads was then performed using FastQC v0.12.1 (*Babraham Bioinformatics—FastQC A Quality Control Tool for High Throughput Sequence Data*, n.d.). Subsequently, processed reads were mapped to their corresponding reference genomes using bwa‐aln v0.7.18 (Li and Durbin 2009) with disabled seed (option‐l 1024). Reference sequences of *O. sativa* , *O. rufipogon* and *H. spontaneum* were downloaded from National Center for Biotechnology Information (NCBI) (GCA_001433935.1, GCA_037997075.1 and GCA_949783245.1, respectively). Unmapped reads were then flagged and removed by using bwa‐samse and samtools v1.21 (Danecek et al. 2021).

#### 2.3.2 Endogenous Content

Endogenous DNA content of each sample was measured by the proportion of the number of mapped reads (primary alignments to its corresponding reference genome without filtering) to the total number of quality‐control (QC)‐passed reads. Samtools flagstat tool (Danecek et al. 2021) was used to extract this information from sam files. Endogenous contents across different tissue types within each species were then plotted using the ggplot2 package in R.

#### 2.3.3 Library Complexity

Library complexity—estimated from the fraction of mapped and unfiltered distinct/unique reads in each sequencing library was estimated by using preseq c‐curve function (Daley and Smith 2013). Due to low endogenous contents and low sequencing depth, most *O. sativa* leaf libraries had less than 20,000 reads post filtering (merging, quality check and mapping), with the lowest library containing ~4000 reads, while the majority of embryo libraries of three examined species as well as leaf libraries of *O. rufipogon* and *H. spontaneum* had at least 480,000 merged mapped reads, with the largest library comprising ~53 million reads. C‐curve complexity analysis showed unstable estimates at the 4000 reads mark and therefore, complexity output was reported and plotted at a fixed depth of 480,000 reads, when available (Supplementary Table 2). For samples where the number of mapped reads was lower than the read threshold, library complexity was reported at the actual read numbers. Finally, PCR optical duplicates were removed by using DeDup v0.12.9 (Peltzer et al. 2016) for downstream analysis.

#### 2.3.4 C‐to‐T Misincorporations

Fractions of C‐to‐T misincorporations of the first 30 nucleotides (5′‐end) were determined by using the AMBER tool (Dolenz et al. 2024) and then visualised using the ggplot2 package in R.

#### 2.3.5 Median Fragment Length

For each sample, read lengths and read counts per length bin were calculated by AMBER tool (Dolenz et al. 2024). As fragment length distributions were typically right‐skewed, with many short fragments and fewer long fragments, a lognormal distribution was fitted to determine the median fragment length as well as standard deviation (SD) value for each sample. This analysis was carried out by using the fitdistr function (MASS package) in R following Weiß et al. (2016). Visualisation of these variations by tissue type was performed using ggplot2 package in R.

#### 2.3.6 Damage Fraction Per Site (*λ*) and Decay Rates (*k*)

The damage fraction per site (*λ*) of each sample was estimated by using a previously described method (Allentoft et al. 2012; Weiß et al. 2016). Briefly, the fragment length having the highest number of reads (peak fragment length) was determined, and all fragment lengths greater than or equal to this peak and up to 110 bp were isolated. This is to avoid the part of the distribution that is affected by the loss of small molecules during laboratory processing. A log‐linear regression model was then fitted to this subset to determine the relationship between the fragment length and the logarithm value of the corresponding fragment frequency. The negative of this regression slope (*λ*) illustrated the fraction of DNA bonds per nucleotide in the sample—the rate at which the number of reads decreased with increasing fragment length. The slope of linear regressions of *λ* versus sample age grouped by tissue type and species was designated as the DNA decay rate (*k*). Other statistical features including *R* ^2^ and *p*‐values were also calculated to evaluate the relationship between DNA degradation and age in accordance with species and tissue characteristics.

## 3 Results

### 3.1 DNA Isolation and Library Preparation

Historical DNA was successfully isolated from leaves and individual seed embryos obtained from herbarium specimens of cultivated rice ( *O. sativa* ), wild rice ( *O. rufipogon* ) and wild barley ( *H. spontaneum* ), spanning approximately 30 to 200 years in age (Table 1). All dehusked grains contained an identifiable embryo that was used for DNA isolation. gDNA concentrations ranged from 0.0015 to 22 ng/μL, corresponding to total yields of 0.15 ng–2.2 μg. No linear relationship was observed between the amount of leaf tissue used and the achieved gDNA yield (Table 1). The mass of embryos was below the detection limit of laboratory scales and was not reported. Regardless of the significantly low gDNA yields of some samples (especially in embryo DNA samples), sufficient amounts of gDNA were isolated for downstream library preparation as well as whole genome sequencing (Table 1). gDNA concentrations for half the negative controls are not detectable and for another half—very low (0.02–0.100 ng/μL) (Supplementary Table 3). In the vast majority of negative controls, the fraction of non‐complex reads mapping to the reference genome is less than 0.04 (Supplementary Table 3). Two cases where endogenous DNA proportion was 0.096 and 0.074, respectively, are most likely due to index hopping during sequencing, as the second sequencing run shows no contamination.

### 3.2 Endogenous Content, Library Complexity, Nucleotide Misincorporations and DNA Fragmentation

To estimate contamination levels and the proportion of endogenous DNA, processed DNA reads were mapped to their corresponding reference genomes. Seed embryos of all species (except for one wild barley sample), as well as leaves of *O. rufipogon* and *H. spontaneum* (except for two wild barley samples), showed high endogenous DNA content (83%–98%). In contrast, endogenous percentages of *O. sativa* leaves were relatively low and variable, ranging from ~1.6%–37%. Accordingly, the endogenous DNA proportions were only significantly higher in *O. sativa* seed embryos compared to leaves (permutation test, *p* = 3.66 × 10^−8^) (Figure 2A).

**FIGURE 2:**
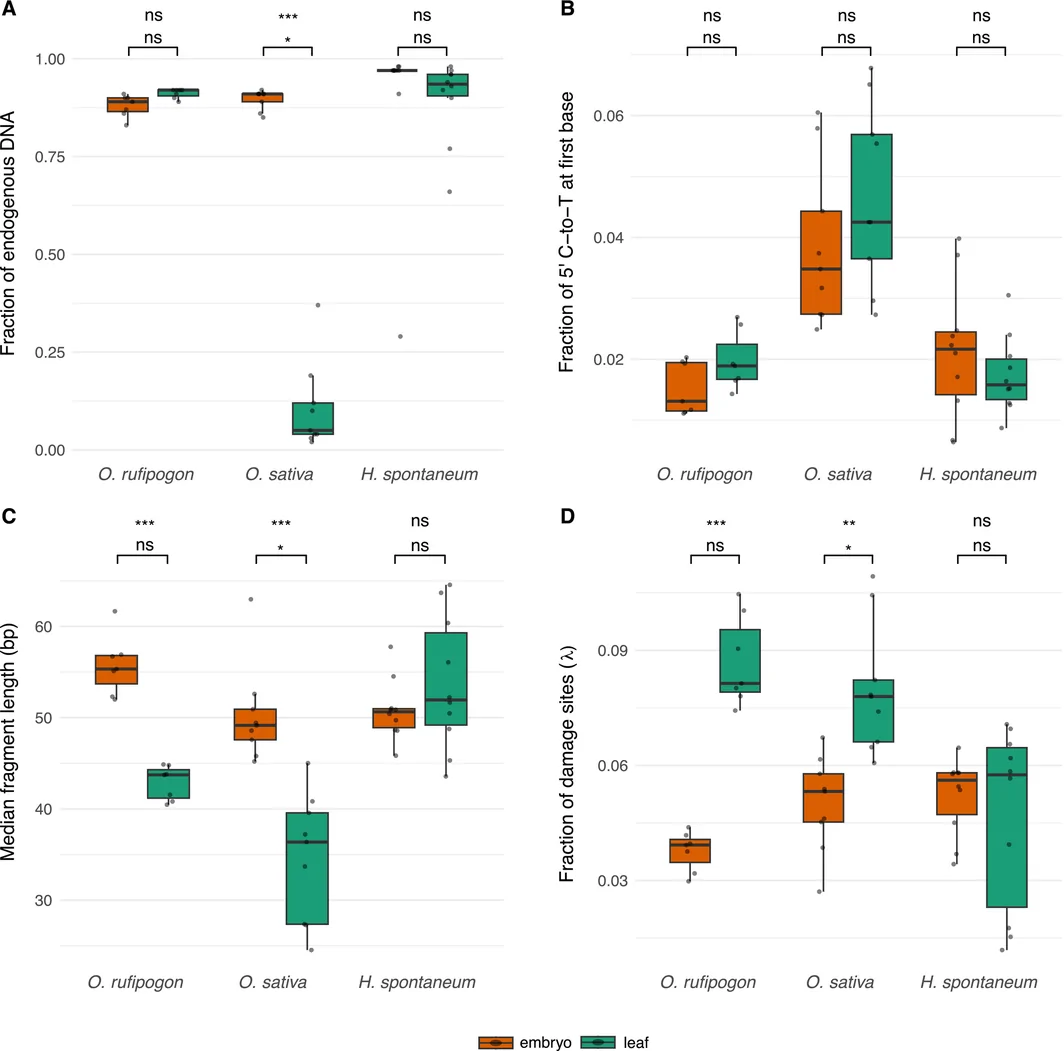
Effects of different tissue types on DNA preservation in historical samples *O. rufipogon* (*N* = 7 pairs), *O. sativa* (*N* = 9 pairs), *H. spontaneum* (*N* = 10 pairs). Box and whisker plots illustrate differences in endogenous content (A), 5′ C‐to‐T fractions at the first base of sequenced reads (B), median fragment length (C) and damage fraction per site (*λ*) (D) of DNA sequencing libraries of wild rice ( *O. rufipogon* ), cultivated rice ( *O. sativa* ) and wild barley ( *H. spontaneum* ). Permutation (below) and paired t‐tests (above) were performed to compare DNA characteristic (A–D) in two tissue types with Bonferroni‐corrected alpha threshold 0.0125 (ns: not significant, *0.0025 ≤ *p* < 0.0125, **0.00025 ≤ *p* < 0.0025, ***p < 0.00025).

Sequenced library complexity was also assessed to validate the capability of very high‐throughput sequencing typical for whole genome sequencing at high coverage for most downstream genomic analyses. Preseq extrapolation curves for both tissue types in *O. rufipogon* and *H. spontaneum* samples indicated that library complexities were consistently high, ranging from 93% to 99%. Cultivated rice *O. sativa* leaf libraries exhibited a wider complexity range (~70% to 96%) (Supplementary Figure 1, Supplementary Table 2).

To evaluate spontaneous post‐mortem modifications (DNA damage), the fraction of first‐base C‐to‐T nucleotide misincorporation—the most common type of miscoding lesions (Briggs et al. 2007), was estimated. Overall, *O. sativa* samples exhibited higher C‐to‐T fractions (up to 0.068) than most samples of the other two species (0.006–0.03). The differences in deamination rates between two tissue types were visible but statistically insignificant across all species based on permutation test (Figure 2B).

Median values, which were estimated from fitted lognormal distributions of merged reads, were used to investigate differences in DNA fragment lengths across tissues and species. Comparisons of these median lengths demonstrated a pronounced tissue‐type effect in *Oryza* samples, with embryo samples showing longer fragments than leaves in *O. sativa* (average difference 18 bp, permutation test, *p* = 3.66 × 10^−3^ at Bonferroni‐corrected *α* = 0.0125) and in *O. rufipogon* (average difference 13 bp, permutation test, *p* = 0.016 at *α* = 0.0125). No visible or significant differences were observed between the two groups in wild barley (Figure 2C).

In conclusion, tissue type had an impact on both the quantity and length of retrieved endogenous DNA from herbarium specimens of examined *Oryza* species, although in *O. rufipogon* these changes were not significant based on permutation test and Bonferroni correction methods. On the other hand, satisfactory yields and quality of endogenous DNA for whole genome sequencing were isolated efficiently from *H. spontaneum* in both tissue types.

Fractions of damage sites (*λ*) were calculated for all samples, followed by permutation tests between two tissue types. The results closely mirrored the results for median fragment length, with significant differences between the tissues in *O. sativa* , non‐significant but visible differences in *O. rufipogon* and no visible nor significant differences in *λ* values in *H. spontaneum* (Figure 2D).

DNA decay rates (*k*) representing the estimated change in *λ* per year were calculated as a regression slope. Across all three species, *k* values of DNA retrieved from leaf tissue were consistently higher than those from corresponding embryo tissue, indicating faster decay rates in leaves, although in many cases the regression correlations between *λ* and age were weak or not statistically significant. In *O. rufipogon* , embryos showed a strong and statistically supported age–damage relationship (*R* ^2^ = 0.825, *p* = 0.005), whereas leaves displayed a weaker correlation (Figure 3A). In *O. sativa* , leaf tissue exhibited both a higher decay rate and a statistically significant positive correlation with age (*k* = 5.86 × 10^−4^, *R* ^2^ = 0.606, *p* = 0.013), while seed embryo tissue had a lower and less consistent decay pattern (Figure 3B). In *H. spontaneum* , decay rates in both tissues were relatively lower (embryo: *k* = 1.05 × 10^−4^ and leaf: *k* = 2.58 × 10^−4^) than those of *Oryza* species and *λ*‐age correlations were weak and not significant, suggesting minimal influence of tissue type on DNA degradation in this species (Figure 3C).

**FIGURE 3:**
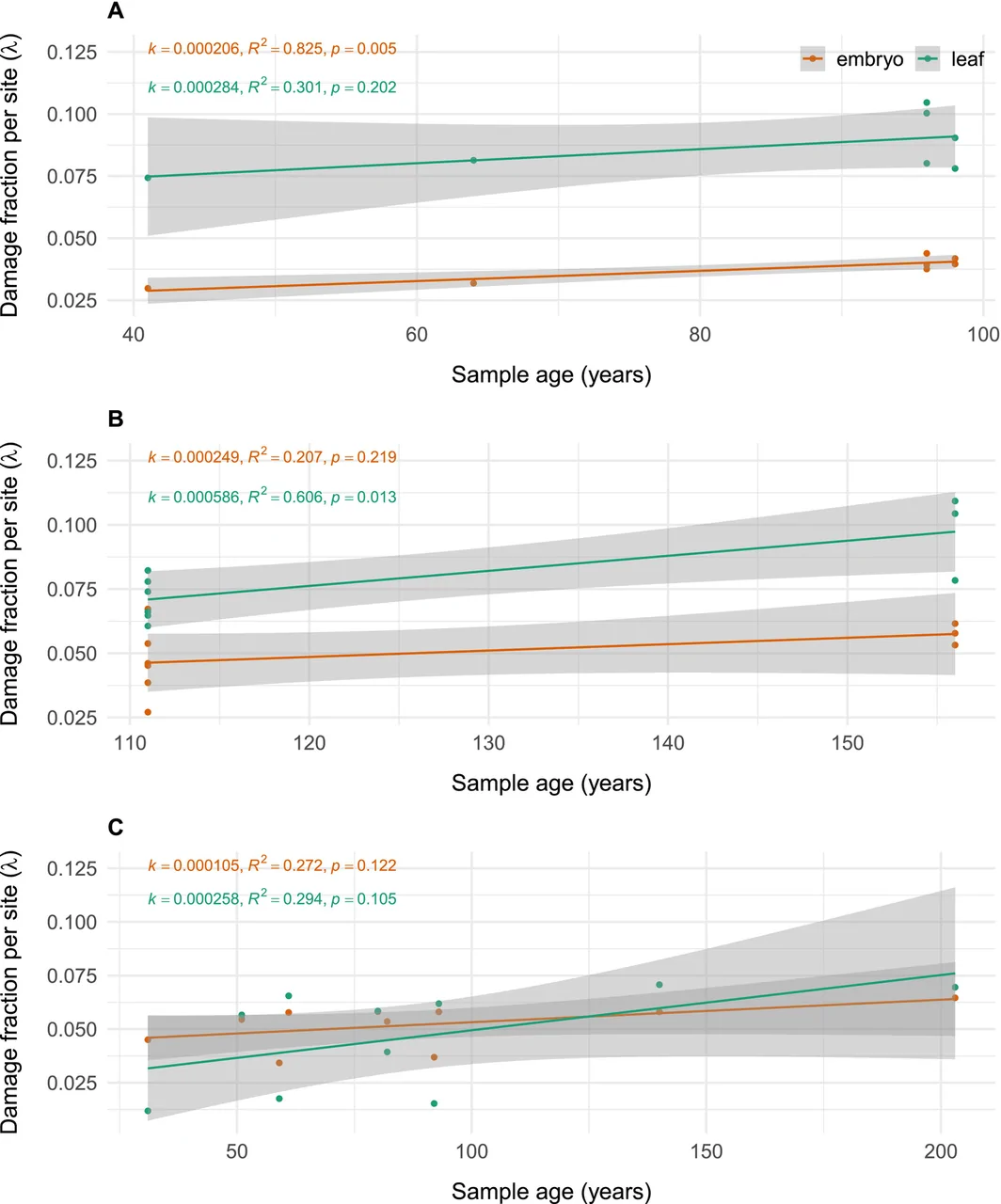
Relationship between damage fraction per site (*λ*) and sample age of DNA. Scatter plots for *λ* values and age in two tissue types across the three investigated species *O. rufipogon* (A), *O. sativa* (B) and *H. spontaneum* (C). The 95% confidence interval was illustrated as grey shaded regions.

Overall, these results demonstrated that tissue type can affect DNA decay rates in investigated *Oryza* samples, with leaves' DNA possibly decaying faster than that of embryos.

### 3.3 Assessment of Historical Crop Seed Materials in Global Collections

Herbarium sheets may have seeds attached to the specimens, in special capsules or in an associated carpological collection. However, seeds are not always present—botanists prefer the flowering stage for most species, which often presents more taxonomy‐informative characteristics. By contrast, biocultural collections usually focus on the utilised portion of the plant, which is the seed or fruit in the case of many crop plants. Many biocultural collections were formed in the eighteenth and nineteenth centuries as research resources in the fields of economic botany and ethnobotany, agronomy, pharmacognosy, food science and others related to useful plants.

Herbarium materials for genetic analysis can be identified either through Index *Herbariorum*, or via the global compendium of specimen records at the *Global Biodiversity Information Facility* (GBIF). By contrast, biocultural collections are usually poorly known and incompletely catalogued, even when stored at the same institutions as herbaria (Cornish and Nesbitt 2014; Nesbitt and Cornish 2016). The Biocultural Collections Group, a specialist network within the Society for Ethnobotany, has done much to bring curators together, but a comprehensive census of such collections is still far from completion (Salick et al. 2014). We draw on our networks to highlight a representative sample of 25 biocultural collections, indicative of those available worldwide. Three largest collections with samples from global distribution are at the Natural History Museum in Paris, the Royal Botanic Gardens, Kew and Vavilov Institute of Plant Industry in Saint Petersburg (Table 2). Most smaller collections offer research resources, more focused on particular crops or geographies (Table 2). Samples in biocultural collections can be housed in herbarium format, attached to paper mounts but emphasising reproductive parts. Alternatively, collections are housed in glass jars, plastic boxes and similar containers (Figure 4).

**TABLE 2:** Representative table of biocultural collections known to have significant holdings of crop seeds.

Collection	Approx. number crops	Date	Specialisms	Country
Powerhouse Museum, Sydney	100+	1850s	Global, Australia	Australia
Economic Botany Collection Meise Botanic Garden	1000+	1821	Global, Africa	Belgium
Canada Agriculture and Food Museum, Ottawa	100+	1800–2000	Canada	Canada
Biocultural Collection Natural History Museum, Copenhagen	500+	1850–1950	Global	Denmark
Ethnobotany collection, National Museum of Natural History, Paris	80,000+	1911–1970s	Southeast Asia and global. Rice, coffee, sorghum, millets.	France
Ethnoecology collection, National Museum of Natural History, Paris	100+	1960s	Global	France
Botanische Gärten der Universität Bonn	1000+	1867–1908	Global. Cereals collection of Friedrich August Körnicke.	Germany
Applied Botany Collection (ABC), Biozentrum Klein Flottbek, University of Hamburg	1000+	1870–1970	Global	Germany
Archaeobotany Laboratory, Bar‐Ilan University	1000+	1870s	Israel and Palestine. Includes wheat landrace collection of Dr. Ludwig Pinner.	Israel
Herbarium, Museo di storia naturale dell'Università degli studi di Firenze	100+	1912–1924	Global, Africa	Italy
Museo agrario tropicale dell'istituto agronomico per Oltremare, Florence	100+	1904–1970s	Africa	Italy
Hideo Hamada collection, Kobe University	1000+	1957–1958	Southeast Asia. Rice.	Japan
Ethnobotany Collection, Institute of Biology, UNAM	100+	1980s	Mexico	Mexico
Economic Botany Collection, Naturalis, Leiden	1000+	1800	Global	Netherlands
Colonial Agricultural Museum, Natural History Museum, Lisbon	100+	1850	Africa	Portugal
Economic Botany Collection, Komarov Botanical Institute, St Petersburg	1000+	1800	Global	Russia
Seed collection, Vavilov Institute of Plant Industry, St Petersburg	10,000+	1890	Global. Distinct from the seedbank collection.	Russia
Botanical Collection, Natural History Museum, University of Zürich	1000+	1850s	Global	Switzerland
Herbarium, Royal Botanic Gardens, Kew.	1000+	20th century	Africa. Snowden collection of sorghum. Also rice, millet etc. These specialist collections with emphasis on crop landraces are held separate to main Herbarium.	United Kingdom
Economic Botany Collection, Royal Botanic Gardens, Kew	10,000+	1847	Global	United Kingdom
John Percival Collection, Herbarium, University of Reading	5000+	< 1921	Eurasia. Wheat. Smaller sets are held by Kew, the Natural History Museum, London and the Vavilov Institute, St Petersburg.	United Kingdom
Timothy C. Plowman Economic Botany Collection, Field Museum, Chicago	1000+	1893	Global, Americas	United States
Biocultural Collection, Missouri Botanical Garden	1000+	1900	Americas. Maize.	United States
Economic Botany Collection, Harvard University Herbaria	1000+	1858–1970s	Global, Americas	United States
Biocultural Collection, New York Botanical Garden	1000+	1891–1970s	Global, Americas	United States

**FIGURE 4:**
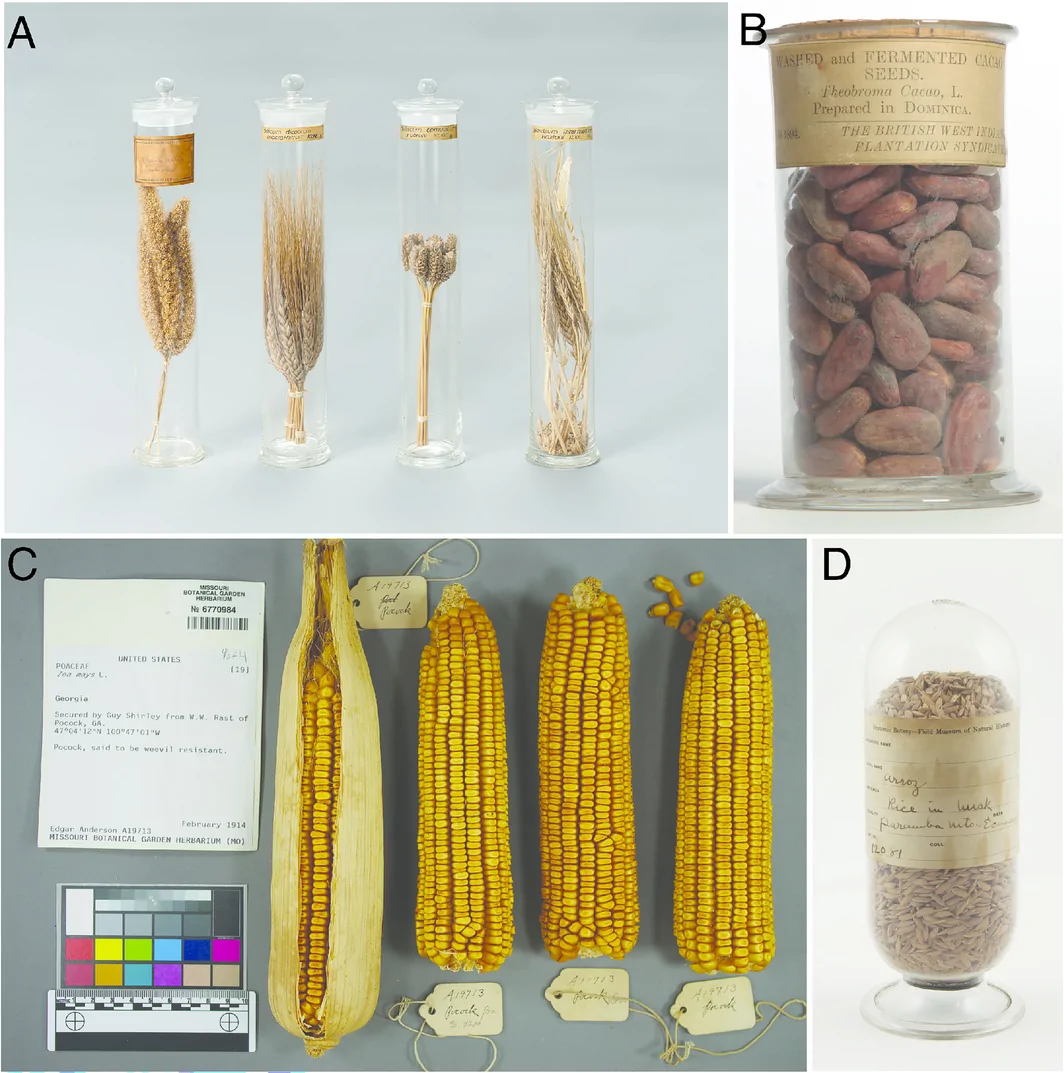
Examples of biocultural collection specimens with crop seeds. Ears of foxtail millet ( *Setaria italica* ), emmer wheat ( *Triticum dicoccum* ), bread wheat ( *Triticum compactum* ); barley (*Hordeum intermedium*), c. 1870, Körnicke collection, University of Bonn (A); CC BY 4.0. Cacao beans, 1894, Dominica, Royal Botanic Gardens, Kew (EBC 070828) (B); CC BY. Yellow dent corn ( *Zea mays* ) from Georgia, USA, 1914, Missouri Botanic Garden (MO‐3056378) (C); CC BY‐NC‐SA 3.0. Rice ( *Oryza sativa* ) grains, Ecuador, Field Museum (Economic Botany 12081) (D); CC BY‐NC 4.0.

## 4 Discussion

The main aim of this study was to test the possibility of whole‐genome sequencing from plant embryos and compare the aDNA characteristics of sequenced libraries generated from embryos to those of leaf tissue across three species, *O. rufipogon* , *O. sativa* and *H. spontaneum* . The results have demonstrated the successful recovery of gDNA from both tissue types, and remarkably, from a single seed embryo of up to ~200 years old specimens. Although gDNA yields were low in some samples, libraries were generated and sequenced efficiently with DNA mixture that would easily allow sequencing whole genomes to a desired depth of coverage. While several previous studies using herbarium materials reported that the DNA inputs of 500 pg to ~10 ng were adequate for genome skimming and targeted enrichment sequencing (Hart et al. 2016; Zeng et al. 2018), sequencing libraries in this study were constructed from as little as ~40 pg of degraded aDNA, providing sufficient quantities for whole‐genome sequencing.

To further evaluate the quality of retrieved aDNA, endogenous content, library complexity, fragment length and damage profiles were estimated. Several previous studies have shown that endogenous DNA proportion was primarily influenced by environmental conditions, including annual precipitation and temperature seasonality, rather than by sample age (Brewer et al. 2019). Differences in tissue properties (e.g., tissue texture, secondary metabolites) approximated by diverse taxonomic groups, also impacted the aDNA preservation in herbarium specimens (Brewer et al. 2019; Kates et al. 2021). Finally, endogenous DNA can be rapidly depleted due to cellular processes and microbial digestion, as well as specimen preparation (e.g., oven‐drying).

Among the three examined species in this study, we represent cereals and grasses with one crop and two crop wild relative species. We also represent tropical (*Oryza*) and temperate (*Hordeum*) environments. *Oryza* species were collected in a wet tropical climate and had similar tissue characteristics. Despite this, we observed very low endogenous DNA content only in *O. sativa* leaf samples, indicating that (i) idiosyncratic processes, such as specimen preparation, differentiated it from *O. rufipogon* leaf samples and (ii) such processes reduced DNA quality in leaf tissue, but not in the embryo. This suggested that the herbarium preparation process in *O. sativa* likely caused substantial DNA loss in leaves, whereas the relatively thick seed husk provided structural protection (Gugerli et al. 2005), preserving endogenous DNA in the embryo. However, the lack of detailed preparation and preservation histories for most herbarium specimens has limited the ability to draw strong conclusions about the impact of processing methods on DNA integrity (Brewer et al. 2019; Staats et al. 2011).

In addition, tissue type effects were observed in both *Oryza* species with respect to the median DNA fragment length. While only the comparison in *O. sativa* was statistically significant, we also observed larger DNA fragments in embryos of *O. rufipogon* . The lack of significance in the latter species is likely an effect of the small sample size. Rapid DNA degradation and fragmentation following organismal death are primarily driven by intracellular nucleases and microbial activities, and these processes are tissue dependent. Subsequently, DNA fragmentation proceeds at a slower, more constant rate, likely caused by depurination (Briggs et al. 2007; Lindahl 1993) and scales with passing time (Porrelli et al. 2026; Weiß et al. 2016). DNA fragmentation is only minimally affected by the climate during collection (Porrelli et al. 2026). The protective effect of the seed coat could have mitigated DNA degradation, resulting in longer median fragment lengths in embryo‐derived sequencing data compared with leaf tissue in tropical *O. sativa* and *O. rufipogon* . Additionally, rice seeds tolerate drying (Choudhary et al. 2023)—the crucial step in herbarium specimen preparation (Bridson and Forman 1998)—and can thus retain seed embryo viability longer than vegetative tissues such as leaves. Consequently, DNA fragmentation and damage may accumulate more slowly in seed embryos than in leaves from the same plant. Conversely, *H. spontaneum* samples collected from temperate environments may have experienced less severe DNA fragmentation in leaf tissue and hence the difference in median length of sequenced DNA compared to embryo is non‐significant. In line with this, the damage fraction per site (*λ*) and its slope (*k*) also differed between tissue types in *Oryza* species. Three *O. sativa* samples have a low number of mapped reads (< 10,000), which might have rendered the calculation of *λ* and *k* less precise. The findings of this study further suggest that the DNA fragmentation in herbarium samples collected from tropics could be partly mitigated by the protective properties of the seed husk, but more data would be necessary to evaluate significance of decay rate differences.

Our study opens up new avenues for research on historical plant materials, particularly crops. Here, we have tested three species (one cultivated and two wild) associated with cereals, the most economically important group of crops. We are optimistic that DNA isolation from embryos of other groups of plants would work well; however, this requires further tests. In particular, we expect that embryos from very small seeds might prove challenging and yield genomic libraries with low complexity. Care needs to be taken as well with seeds that contain abundant secondary metabolites; these, however, often accumulate in endosperm and not in embryos. Precision of dissection might play a pivotal role in such instances.

Herbaria are hugely promising sources of study materials for plant evolution and ecology; however, herbarium specimens have limitations: the amount of plant material available for sampling may be limited, crops are typically under‐collected by botanists, and specimens often do not bear cultural data such as the name of the landrace or cultivar. Conversely, specimens in biocultural collections offer significant advantages to genetics researchers. The emphasis is on the utilised part of the plant, thus seeds and fruits in the case of many crops (Figure 4). They are often in bulk, enabling easier decision making on destructive sampling and often bear significant cultural data.

These specimens will benefit from significant investment in researching provenance and connections to previous archives and publications, which will require advice from curators or historians of science. Compared to genebank collections, seeds in biocultural collections are usually no longer viable, but have typically been stored in conditions that preserve aDNA in reasonable condition. Increased interest in sustainability and plant‐human relations has led to a revival of interest in historic collections, and the formation of new ones (Salick et al. 2014). The approach reported in this study will facilitate the utilisation of genetic data from these rich and unique repositories.

Researchers will need to be creative in searching for such resources. They should bear in mind that the scope of historic collections is often wider than expected: for example, pharmacists usually had to study a wide range of food, dye and textile plants so these are well represented in *materia medica* collections. Art or historical museums may hold substantial biocultural collections deriving from past World's Fairs. We recommend that researchers begin with local collections and make wide enquiries. Use of such collections is key to increased societal value and, thus, increased investment in their curation. Further advice on their use is given by (Allasi Canales et al. 2022).

Application of our approach to ethnobotanical collections opens exceptional opportunities for studying plant evolution, including the creation and management of genetic diversity by farmers. However, care needs to be taken to ensure their legal and ethical utilisation. Crops, medicines, forages and plants with other uses are by definition linked to people, including Indigenous Peoples and local communities. In many cases historic plant specimens embody spiritual beliefs, diverse forms of ownership, traditional knowledge and past histories (Das and Lowe 2018; Hart et al. 2026). Legal elements of the Convention on Biological Diversity (CBD), Nagoya Protocol and Access and Benefit Sharing guidelines should be implemented (Dhanda et al. 2026; Iob and Botigué 2022). Regardless, researchers should seek collaborations with stewards and owners of local useful plants, where possible, engaging them in the project design and outputs from an early point.

In conclusion, this study has demonstrated the feasibility of isolating DNA and constructing sequencing libraries from single seed embryos of *O. sativa* , *O. rufipogon* and *H. spontaneum* . In the genus *Oryza*, embryos consistently outperformed leaves across several measures, whereas in wild barley both tissues delivered satisfactory results without significant differences. Embryos represent a promising alternative source for DNA isolation from herbarium specimens, especially where collection, preparation and processing possibly severely compromise endogenous DNA quality. These findings highlight the broader potential for applying single‐seed embryo DNA isolation in herbaria, particularly when leaf tissue is limited. Additionally, this research opens up rich but previously underutilised historical resources—including seed banks, economic botany collections, anthropological museums and other collections preserving crop grains—for genomic investigation.

## Author Contributions

K.N. and R.M.G. conceived the project; H.P.L. and R.M.G. designed the study and the sampling strategy, with contributions from S.P., Y.‐K.L. S.J., F.P. and K.N.; H.P.L., S.P., S.J. and F.P. curated and sampled herbaria; H.P.L. and S.P. performed the aDNA laboratory work; H.P.L. processed and analysed sequencing data. M.N. compiled and curated the available biocultural collection resources data. H.P.L., R.M.G. and M.N. wrote the manuscript with contributions from all authors.

## Funding

H.P.L., Y.‐K.L. and R.M.G. were funded by UKRI Frontier Research Guarantee (EP/X022404/1) to R.M.G. This work was also supported by the Research Computing at the James Hutton Institute through grants BB/S019669/1 and BB/X019683/1.

## Ethics Statement

No animal materials were used for this study. No plant material used in this study is considered rare, threatened or endangered. This research was conducted in compliance with the Convention on Biological Diversity (CBD) and the Nagoya Protocol on Access and Benefit‐Sharing. All data are available in public repositories.

## Conflicts of Interest

The authors declare no conflicts of interest.

## Supporting information


**Supplementary Figure 1** Effects of different tissue types on the sequenced genomic library complexity in historical samples. Box and whisker plots illustrate the estimated fraction of unique reads in the library. The size of the circles is scaled to the number of mapped reads for which complexity was calculated.

**Supplementary Table 1** Details of herbarium samples (NMNH: National Museum of Natural History, France; RBGK: Royal Botanic Gardens, Kew, United Kingdom).
**Supplementary Table 2**. Details of sequencing data generated for leaf and embryo tissue. NA—not applicable, owing to too few mapped reads.
**Supplementary Table 3**. Mapping statistics of negative controls for DNA extraction (extraction blank, EB) and library preparation (library blank, LB). NA, not applicable, negative controls for library preparation use water rather than DNA isolate.

## Data Availability

Raw FASTQ DNA sequences of all samples are deposited on the sequence reads archive (SRA) on NCBI under BioProject PRJNA1429617. Individual accessions for each sample are provided in Supplementary Table 2.

## References

[men70210-bib-0001] Allasi Canales, N. , A. Clarke , M. Nesbitt , and R. Gutaker . 2022. “DNA From Museum Collections.” In Molecular Identification of Plants: From Sequence to Species, 29–42. Advanced Books, Pensoft Publishers. 10.3897/ab.e98875.

[men70210-bib-0002] Allentoft, M. E. , M. Collins , D. Harker , et al. 2012. “The Half‐Life of DNA in Bone: Measuring Decay Kinetics in 158 Dated Fossils.” Proceedings of the Royal Society B: Biological Sciences 279, no. 1748: 4724–4733. 10.1098/rspb.2012.1745. PMC3497090 23055061

[men70210-bib-0003] Babraham Bioinformatics . n.d. “FastQC A Quality Control Tool for High Throughput Sequence Data.” https://www.bioinformatics.babraham.ac.uk/projects/fastqc/.

[men70210-bib-0004] Bennett, M. D. , J. B. Smith , and I. Barclay . 1975. “Early Seed Development in the Triticeae.” Philosophical Transactions of the Royal Society of London. B, Biological Sciences 272, no. 916: 199–227. 10.1098/rstb.1975.0083.

[men70210-bib-0005] Brewer, G. E. , J. J. Clarkson , O. Maurin , et al. 2019. “Factors Affecting Targeted Sequencing of 353 Nuclear Genes From Herbarium Specimens Spanning the Diversity of Angiosperms.” Frontiers in Plant Science 10: 477275. 10.3389/fpls.2019.01102. PMC6759688 31620145

[men70210-bib-0006] Bridson, D. M. , and L. Forman . 1998. The Herbarium Handbook. 3rd ed. Royal Botanic Gardens, Kew.

[men70210-bib-0007] Briggs, A. W. , U. Stenzel , P. L. F. Johnson , et al. 2007. “Patterns of Damage in Genomic DNA Sequences From a Neandertal.” Proceedings of the National Academy of Sciences 104, no. 37: 14616–14621. 10.1073/pnas.0704665104. PMC1976210 17715061

[men70210-bib-0008] Burbano, H. A. , and R. M. Gutaker . 2023. “Ancient DNA Genomics and the Renaissance of Herbaria.” Science 382, no. 6666: 59–63. 10.1126/science.adi1180. 37797028

[men70210-bib-0009] Choudhary, P. , L. Pramitha , P. R. Aggarwal , S. Rana , M. Vetriventhan , and M. Muthamilarasan . 2023. “Biotechnological Interventions for Improving the Seed Longevity in Cereal Crops: Progress and Prospects.” Critical Reviews in Biotechnology 43, no. 2: 309–325. 10.1080/07388551.2022.2027863. 35443842

[men70210-bib-0010] Cornish, C. , and M. Nesbitt . 2014. “Historical Perspectives on Western Ethnobotanical Collections. Chapter.” 20: 271–294.

[men70210-bib-0011] Daley, T. , and A. D. Smith . 2013. “Predicting the Molecular Complexity of Sequencing Libraries.” Nature Methods 10, no. 4: 325–327. 10.1038/nmeth.2375. PMC3612374 23435259

[men70210-bib-0012] Danecek, P. , J. K. Bonfield , J. Liddle , et al. 2021. “Twelve Years of SAMtools and BCFtools.” GigaScience 10, no. 2: giab008–giab008. 10.1093/gigascience/giab008. PMC7931819 33590861

[men70210-bib-0013] Das, S. , and M. Lowe . 2018. “Nature Read in Black and White: Decolonial Approaches to Interpreting Natural History Collections.” Journal of Natural Science Collections 6, no. 4: 4–14.

[men70210-bib-0014] Dhanda, S. , E. Lee , T. Livermore , et al. 2026. “Exploring the Legal, Policy, Ethical and Practical Implications of Digitisation of Botanical and Fungal Collections.” Plants, People, Planet 8, no. 4: 1076–1086. 10.1002/ppp3.70227.

[men70210-bib-0015] Dolenz, S. , T. van der Valk , C. Jin , et al. 2024. “Unravelling Reference Bias in Ancient DNA Datasets.” Bioinformatics 40, no. 7: btae436–btae436. 10.1093/bioinformatics/btae436. PMC11254355 38960861

[men70210-bib-0016] Drábková, L. Z. 2013. “DNA Extraction From Herbarium Specimens.” In Molecular Plant Taxonomy: Methods and Protocols, 69–84. Springer. 10.1007/978-1-62703-767-9_4. 24415470

[men70210-bib-0017] Gros‐Balthazard, M. , J. M. Flowers , K. M. Hazzouri , et al. 2021. “The Genomes of Ancient Date Palms Germinated From 2,000 y Old Seeds.” Proceedings of the National Academy of Sciences of the United States of America 118, no. 19: e2025337118–e2025337118. 10.1073/pnas.2025337118. PMC8126781 33941705

[men70210-bib-0018] Gugerli, F. , L. Parducci , and R. J. Petit . 2005. “Ancient Plant DNA: Review and Prospects.” New Phytologist 166, no. 2: 409–418. 10.1111/j.1469-8137.2005.01360.x. 15819905

[men70210-bib-0019] Gutaker, R. M. , and H. A. Burbano . 2017. “Reinforcing Plant Evolutionary Genomics Using Ancient DNA.” Current Opinion in Plant Biology 36: 38–45. 10.1016/j.pbi.2017.01.002. 28160617

[men70210-bib-0020] Gutaker, R. M. , E. Reiter , A. Furtwängler , V. J. Schuenemann , and H. A. Burbano . 2017. “Extraction of Ultrashort DNA Molecules From Herbarium Specimens.” BioTechniques 62, no. 2: 76–79. 10.2144/000114517. 28193151

[men70210-bib-0021] Hagenblad, J. , J. Morales , M. W. Leino , R. Abbey‐Lee , A. C. Rodríguez‐Rodríguez , and J. Santana . 2024. “Utilising Ancient DNA to Understand Crop Population Dynamics Across a Millennium: A Case Study of Archaeological Barley ( *Hordeum vulgare* L.) From Gran Canaria, Spain.” Journal of Archaeological Science 167: 106001–106001. 10.1016/j.jas.2024.106001.

[men70210-bib-0022] Hart, M. L. , L. L. Forrest , J. A. Nicholls , and C. A. Kidner . 2016. “Retrieval of Hundreds of Nuclear Loci From Herbarium Specimens.” Taxon 65, no. 5: 1081–1092. 10.12705/655.9.

[men70210-bib-0023] Hart, R. , V. Fonseca‐Kruel , E. Dalcin , et al. 2026. “Repositories of Biocultural Diversity: Toward Best Practices for Empowering Ethnobotany in Digital Herbaria.” Plants, People, Planet 8, no. 4: 1095–1103. 10.1002/ppp3.70052.

[men70210-bib-0024] Heberling, J. M. , L. A. Prather , and S. J. Tonsor . 2019. “The Changing Uses of Herbarium Data in an Era of Global Change: An Overview Using Automated Content Analysis.” Bioscience 69, no. 10: 812–822. 10.1093/biosci/biz094.

[men70210-bib-0025] Hiraoka, T. , N. Ikeda , R. Ishikawa , and T. Ishii . 2009. “Evaluation of Genetic Diversity Among Rice Local Varieties in Vietnam.” Journal of Crop Research 54: 125–130.

[men70210-bib-0026] Index Herbariorum . n.d. “The William & Lynda Steere Herbarium.” https://sweetgum.nybg.org/science/ih/.

[men70210-bib-0027] Iob, A. , and L. Botigué . 2022. “Crop Archaeogenomics: A Powerful Resource in Need of a Well‐Defined Regulation Framework.” Plants, People, Planet 4, no. 1: 44–50. 10.1002/ppp3.10233.

[men70210-bib-0028] Itoh, J.‐I. , K.‐I. Nonomura , K. Ikeda , et al. 2005. “Rice Plant Development: From Zygote to Spikelet.” Plant and Cell Physiology 46, no. 1: 23–47. 10.1093/pcp/pci501. 15659435

[men70210-bib-0029] Kates, H. R. , J. R. Doby , C. M. Siniscalchi , et al. 2021. “The Effects of Herbarium Specimen Characteristics on Short‐Read NGS Sequencing Success in Nearly 8000 Specimens: Old, Degraded Samples Have Lower DNA Yields but Consistent Sequencing Success.” Frontiers in Plant Science 12: 669064–669064. 10.3389/fpls.2021.669064. PMC8262526 34249041

[men70210-bib-0030] Kircher, M. , S. Sawyer , and M. Meyer . 2012. “Double Indexing Overcomes Inaccuracies in Multiplex Sequencing on the Illumina Platform.” Nucleic Acids Research 40, no. 1: e3–e3. 10.1093/nar/gkr771. PMC3245947 22021376

[men70210-bib-0031] Kistler, L. , V. C. Bieker , M. D. Martin , M. W. Pedersen , J. Ramos Madrigal , and N. Wales . 2020. “Ancient Plant Genomics in Archaeology, Herbaria, and the Environment.” Annual Review of Plant Biology 71, no. 1: 605–629. 10.1146/annurev-arplant-081519-035837. 32119793

[men70210-bib-0032] Kistler, L. , R. Ware , O. Smith , M. Collins , and R. G. Allaby . 2017. “A New Model for Ancient DNA Decay Based on Paleogenomic Meta‐Analysis.” Nucleic Acids Research 45, no. 11: 6310–6320. 10.1093/nar/gkx361. PMC5499742 28486705

[men70210-bib-0033] Kobayashi, A. , K. Ebana , S. Fukuoka , and T. Nagamine . 2006. “Microsatellite Markers Revealed the Genetic Diversity of an Old Japanese Rice Landrace ‘Echizen’.” Genetic Resources and Crop Evolution 53, no. 3: 499–506. 10.1007/s10722-004-2029-z.

[men70210-bib-0034] Latorre, S. M. , P. L. M. Lang , H. A. Burbano , and R. M. Gutaker . 2020. “Isolation, Library Preparation, and Bioinformatic Analysis of Historical and Ancient Plant DNA.” Current Protocols in Plant Biology 5, no. 4: e20121–e20121. 10.1002/cppb.20121. 33211414

[men70210-bib-0035] Leino, M. W. , J. Hagenblad , J. Edqvist , and E.‐M. K. Strese . 2009. “DNA Preservation and Utility of a Historic Seed Collection.” Seed Science Research 19, no. 3: 125–135. 10.1017/S0960258509990055.

[men70210-bib-0036] Li, H. , and R. Durbin . 2009. “Fast and Accurate Short Read Alignment With Burrows–Wheeler Transform.” Bioinformatics 25, no. 14: 1754–1760. 10.1093/bioinformatics/btp324. PMC2705234 19451168

[men70210-bib-0037] Liang, H. , Y. Deng , C. Wang , and X. Xu . 2016. “A High‐Throughput DNA Extraction Method From Rice Seeds.” Biotechnology & Biotechnological Equipment 30, no. 1: 32–35. 10.1080/13102818.2015.1088401.

[men70210-bib-0038] Lindahl, T. 1993. “Instability and Decay of the Primary Structure of DNA.” Nature 362, no. 6422: 709–715. 10.1038/362709a0. 8469282

[men70210-bib-0039] Lister, D. L. , S. Thaw , M. A. Bower , et al. 2009. “Latitudinal Variation in a Photoperiod Response Gene in European Barley: Insight Into the Dynamics of Agricultural Spread From ‘Historic’ Specimens.” Journal of Archaeological Science 36, no. 4: 1092–1098. 10.1016/j.jas.2008.12.012.

[men70210-bib-0040] Mascher, M. , V. J. Schuenemann , U. Davidovich , et al. 2016. “Genomic Analysis of 6,000‐Year‐Old Cultivated Grain Illuminates the Domestication History of Barley.” Nature Genetics 48, no. 9: 1089–1093. 10.1038/ng.3611. 27428749

[men70210-bib-0041] Meyer, M. , and M. Kircher . 2010. “Illumina Sequencing Library Preparation for Highly Multiplexed Target Capture and Sequencing.” Cold Spring Harbor Protocols 2010, no. 6: pdb‐prot5448. 10.1101/pdb.prot5448. 20516186

[men70210-bib-0042] Muto, C. , K. Tanaka , H. Tabuchi , N. Kurauchi , Y.‐I. Sato , and R. Ishikawa . 2020. “Genetic Diversity of Preserved Rice Seed Samples From the Mikawa Area, Japan, Stored in the Meiji Era.” Breeding Science 70, no. 3: 347–354. 10.1270/jsbbs.19143. PMC7372032 32714057

[men70210-bib-0043] Nesbitt, M. , and C. Cornish . 2016. “Seeds of Industry and Empire: Economic Botany Collections Between Nature and Culture.” Journal of Museum Ethnography 29: 53–70.

[men70210-bib-0044] Ng, S.‐K. , and R. M. Gutaker . 2025. “Negligible Effect of Host DNA on Metagenomic Analysis Enables Microbial Ecology Investigation in Historical Samples.” bioRxiv: The Preprint Server for Biology 2024–2025: 647358. 10.1101/2025.04.05.647358.

[men70210-bib-0045] Palmer, S. A. , J. D. Moore , A. J. Clapham , P. Rose , and R. G. Allaby . 2009. “Archaeogenetic Evidence of Ancient Nubian Barley Evolution From Six to Two‐Row Indicates Local Adaptation.” PLoS One 4, no. 7: e6301. 10.1371/journal.pone.0006301. PMC2707625 19623249

[men70210-bib-0046] Peltzer, A. , G. Jäger , A. Herbig , et al. 2016. “EAGER: Efficient Ancient Genome Reconstruction.” Genome Biology 17, no. 1: 60–60. 10.1186/s13059-016-0918-z. PMC4815194 27036623

[men70210-bib-0047] Percival‐Alwyn, L. , I. Barnes , M. D. Clark , et al. 2025. “UKCropDiversity‐HPC: A Collaborative High‐Performance Computing Resource Approach for Sustainable Agriculture and Biodiversity Conservation.” Plants, People, Planet 7, no. 4: 969–977. 10.1002/ppp3.10607.

[men70210-bib-0048] Porrelli, S. , A. Fornasiero , H. P. Le , et al. 2026. “Patterns of aDNA Damage Through Time and Environments—Lessons From Herbarium Specimens.” GigaScience 15: giag026. 10.1093/gigascience/giag026. PMC13108254 41784526

[men70210-bib-0049] Quatela, A.‐S. , P. Cangren , F. Jafari , T. Michel , H. J. De Boer , and B. Oxelman . 2023. “Retrieval of Long DNA Reads From Herbarium Specimens.” AoB Plants 15, no. 6: plad074. 10.1093/aobpla/plad074. PMC10735254 38130422

[men70210-bib-0050] Rogers, S. O. , and A. J. Bendich . 1985. “Extraction of DNA From Milligram Amounts of Fresh, Herbarium and Mummified Plant Tissues.” Plant Molecular Biology 5, no. 2: 69–76. 10.1007/BF00020088. 24306565

[men70210-bib-0051] Salick, J. , K. Konchar , and M. Nesbitt . 2014. Curating Biocultural Collections: A Handbook. Kew Publishing.

[men70210-bib-0052] Särkinen, T. , M. Staats , J. E. Richardson , R. S. Cowan , and F. T. Bakker . 2012. “How to Open the Treasure Chest? Optimising DNA Extraction From Herbarium Specimens.” PLoS One 7, no. 8: e43808. 10.1371/journal.pone.0043808. PMC3429509 22952770

[men70210-bib-0053] Schubert, M. , S. Lindgreen , and L. Orlando . 2016. “AdapterRemoval v2: Rapid Adapter Trimming, Identification, and Read Merging.” BMC Research Notes 9, no. 1: 88–88. 10.1186/s13104-016-1900-2. PMC4751634 26868221

[men70210-bib-0054] Staats, M. , A. Cuenca , J. E. Richardson , et al. 2011. “DNA Damage in Plant Herbarium Tissue.” PLoS One 6, no. 12: e28448–e28448. 10.1371/journal.pone.0028448. PMC3230621 22163018

[men70210-bib-0055] Sugita, N. , A. Ebihara , T. Hosoya , et al. 2020. “Non‐Destructive DNA Extraction From Herbarium Specimens: A Method Particularly Suitable for Plants With Small and Fragile Leaves.” Journal of Plant Research 133, no. 1: 133–141. 10.1007/s10265-019-01152-4. 31760570

[men70210-bib-0056] Walters, C. , A. A. Reilley , P. A. Reeves , J. Baszczak , and C. M. Richards . 2006. “The Utility of Aged Seeds in DNA Banks.” Seed Science Research 16, no. 3: 169–178. 10.1079/SSR2006246.

[men70210-bib-0057] Weiß, C. L. , V. J. Schuenemann , J. Devos , et al. 2016. “Temporal Patterns of Damage and Decay Kinetics of DNA Retrieved From Plant Herbarium Specimens.” Royal Society Open Science 3, no. 6: 160239–160239. 10.1098/rsos.160239. PMC4929915 27429780

[men70210-bib-0058] Zeng, C.‐X. , P. M. Hollingsworth , J. Yang , et al. 2018. “Genome Skimming Herbarium Specimens for DNA Barcoding and Phylogenomics.” Plant Methods 14, no. 1: 43–43. 10.1186/s13007-018-0300-0. PMC5987614 29928291

