# Supplementary material for: Historical Plant Embryos as Alternative Sources of Ancient DNA for Whole Genome Sequencing
Source: Mol Ecol Resour. 2026 Oct 7;26(7):e70210. doi: 10.1111/1755-0998.70210 (PMC13646664; doi:10.1111/1755-0998.70210)
Supplement: Supplementary file 1 [file MEN-26-e70210-s002.pdf]

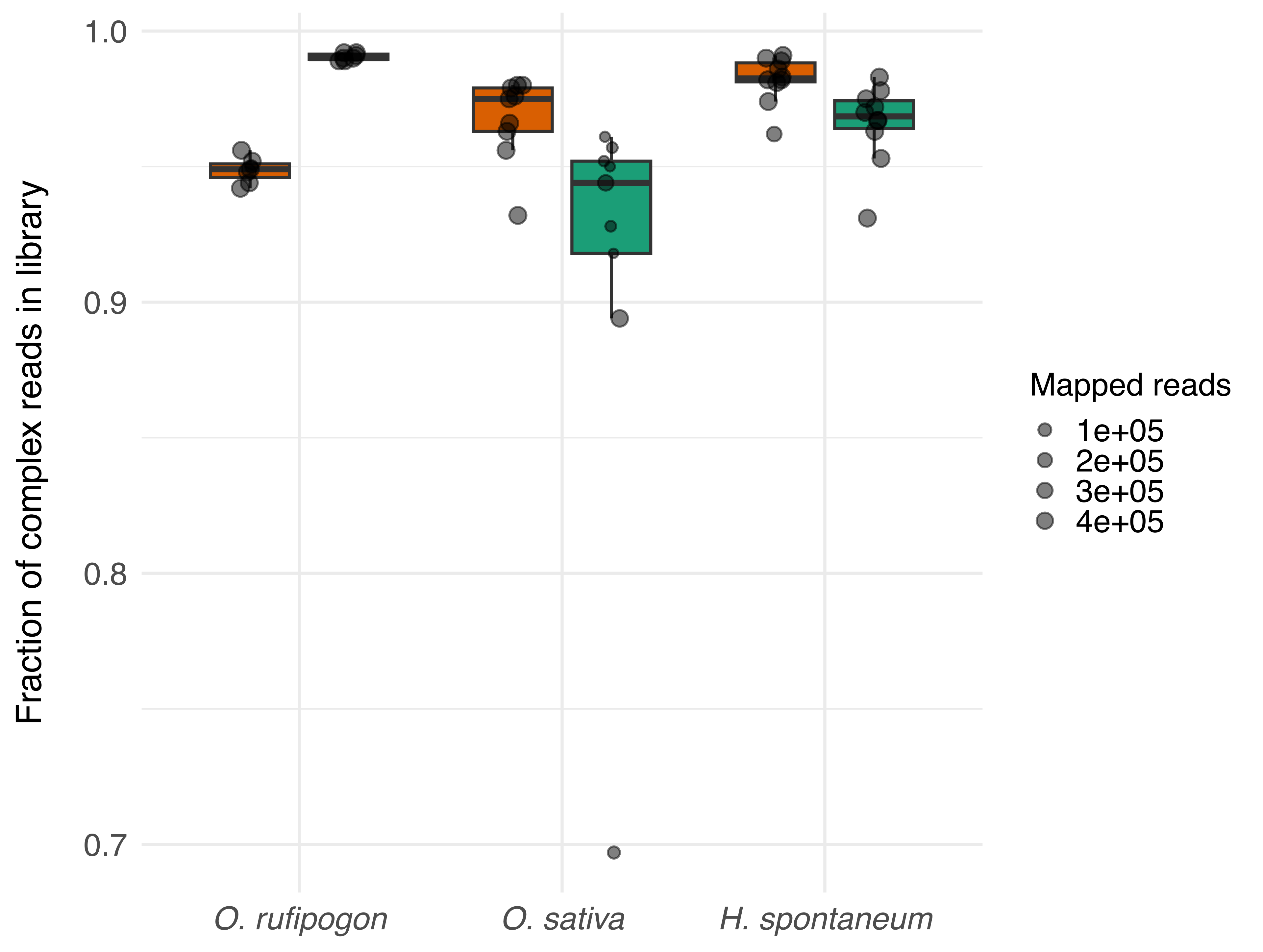

Fraction of complex reads in library
1.0
0.9
0.8
0.7
O. rufipogon
O. sativa
H. spontaneum
Mapped reads
1e+05
2e+05
3e+05
4e+05
